# The role of Wnt signaling pathway in carcinogenesis and implications for anticancer therapeutics

**DOI:** 10.1186/1897-4287-12-13

**Published:** 2014-04-22

**Authors:** Asfandyar Sheikh, Asfandyar Khan Niazi, Muhammad Zafar Ahmed, Bushra Iqbal, Syed Muhammad Saad Anwer, Hira Hussain Khan

**Affiliations:** 1 Dow Medical College, Dow University of Health Sciences, Baba-e-Urdu Road, 74200 Karachi, Pakistan; 2 Shifa College of Medicine, H-8/4 Islamabad, Islamabad, Pakistan; 3 Jinnah Medical and Dental College, Karachi, Pakistan

## Abstract

The Wnt proteins are a family of 19 secreted glycoproteins that occupy crucial roles in the regulation of processes such as cell survival, proliferation, migration and polarity, cell fate specification, body axis patterning and self-renewal in stem cells. The canonical pathway has been implicated in a variety of cancers. As such, it is only fair to conclude that therapies targeting the Wnt pathway may play an essential role in the future of anticancer therapeutics, both alone or in conjunction with traditional therapies.

## Article

The Wnt signaling pathways represent a group of pathways that comprise of proteins involved in the transduction of signals via cell surface receptors. First identified in 1982 by Nusse in mouse models of mammary cancer, these pathways can be broadly divided into two major groups: canonical and non-canonical, with the differentiating factor being the involvement of β-catenin (encoded by CTNNB1) in the former [1,2]. Both are activated by the binding of a Wnt-protein ligand to a Frizzled family receptor (Fz), which in turn transfers the signal to the intracellular protein, Dishevelled (Dsh) [3].

The Wnt proteins are a family of 19 secreted glycoproteins that are 350–400 amino acids in length [4]. They occupy crucial roles in the regulation of processes such as cell survival, proliferation, migration and polarity, cell fate specification, body axis patterning and self-renewal in stem cells [5]. Mutations of genes involved in this pathway may lead to alteration of the activities of the proteins necessary for signal transduction, a phenomenon that may lead to defects in embryonic development, or may result in a multitude of diseases (e.g. type II diabetes and late onset Alzheimer) in adults [6].

**Figure 1:**
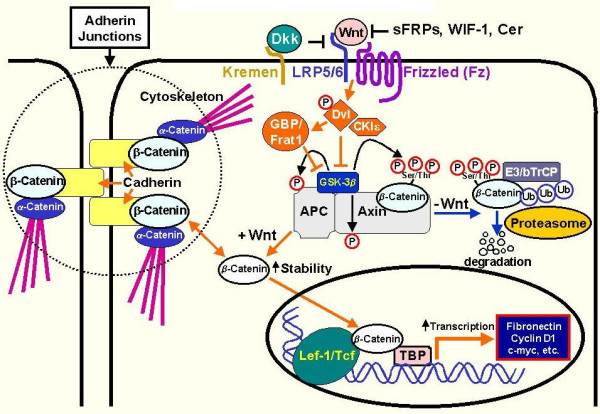
**An overview of the canonical Wnt pathway [**[11]**].**

While the non-canonical pathways are involved in functions such as cytoskeleton development and intracellular calcium homeostasis, the canonical pathway (Figure 1) has greater implications for tumorigenesis [7]. The hallmark protein for the canonical pathway, β-catenin, plays a role in the activation of transcription factors belonging to the TCF/LEF family [8]. In the absence of Wnt signaling, β-catenin fails to accumulate in the cytoplasm due to destruction by a degradation complex comprising proteins such as APC, axin, PP2A, GSK3, CK1α and WTX [8]. Binding of Wnt to Fz and LRP-5/6 causes disruption of the degradation complex, thereby leading to accumulation of β-catenin [9]. As such, inappropriate activation of the canonical pathway may lead to elevated levels of intracellular β-catenin. This may occur as a result of mutations in β-catenin or other proteins in the pathway, overexpression of Wnt ligands and/or loss of inhibitors or regulatory proteins [10].

The earliest evidence of Wnt involvement in human cancers was with the discovery of the association between APC tumor suppressor gene and β-catenin [12]. Loss of function mutations in APC are involved in familial adenomatous polyposis, a heritable cancer syndrome, and various forms of sporadic colorectal cancers [13]. Other mutations include those involving axins I and II, both of which are tumor suppressors involved in the downregulation of β-catenin [14].

Inappropriate Wnt signaling has also been implicated in various facets of both benign and malignant breast tumors [15]. Elevated levels of β-catenin in breast cancer were first demonstrated by Lin et al. in 2000 [16]. The increased levels were found to correlate with increased expression of cyclin D1 [16]. Wnt involvement in the epithelial-mesenchymal transition has also been observed in metastasis of basal-like breast cancer to the lungs [17]. Similarly, increased β-catenin levels are also detected in melanomas [18]. Inhibition of beta-catenin in metastatic melanoma cell lines has been shown to induce apoptosis, inhibit proliferation, migration and invasion, and decrease chemoresistance [19].

Hepatocellular carcinoma (HCC) has also been identified as a heterogeneous cancer with active Wnt signaling [20]. The dysregulation of Wnt signaling in HCC has been attributed to two distinct molecular classes, namely CTNNB1 and Wnt-TGFβ [21]. Recent evidence suggests that Glypican-3 (GPC3), a cell surface heparan sulfate proteoglycan, is highly expressed in HCC and may stimulate HCC growth by stabilizing the interaction between Wnt and Fz, thereby causing activation of downstream pathways [22]. Similarly, wnt inhibition has also been shown to inhibit proliferation and induce apoptosis of cultured pancreatic adenocarcinoma (PAC) cells [23]. Wnt signaling may also play a role in drug resistance in PAC via mechanisms such as angiogenesis, highly resistant cancer stem cells or dysregulation of cell cycle and apoptosis [24].

Inappropriate Wnt activation is also critical in certain lung cancers [25]. At least three mechanisms have been identified, namely, overexpression of Wnt effectors such as Dvl, activation of a non-canonical pathway involving JNK and repression of Wnt antagonists such as WIF-1 [25]. Wnt activation has also been implicated in neuronal differentiation in glioblastoma and angiogenesis in gliomas [26,27].

Dysregulation of the canonical pathway in endometrial carcinoma can be attributed to inactivating β-catenin mutations or downregulation of Wnt antagonists by epigenetic silencing [28]. The wnt pathway is additionally involved with estrogen and progesterone, which further elucidates its significance [28]. Activation of the canonical pathway also exercises effects on prostate cell proliferation, differentiation and epithelial-mesenchymal transition [29]. Elevated levels of β-catenin have been implicated in prostate cancer progression, due to its association with the androgen receptor [30]. Recent studies have also identified a role of Wnt pathway in Wilm’s tumor [31,32].

In view of the above discussion, it is only fair to conclude that therapies targeting the Wnt pathway may play an essential role in the future of anticancer therapeutics, both alone or in conjunction with traditional therapies. Recent reports have grouped potential Wnt inhibitors into three major categories: small molecules, antibodies and peptides [33]. The first category includes low molecular weight compounds that modulate Wnt signaling, in vivo. For example, NSAIDs and celecoxib have been shown to inhibit CTNNB1-dependent transcription in colorectal cells [34,35]. XAV939 and pyrvinium are two novel compounds falling in this category [36,37]. XAV939 inhibits tankyrase thereby stabilizing AXIN, whereas pyrvinium upregulates CTNNB1 phosphorylation via activation of casein kinase [36,37]. The second category includes blocking antibodies that decrease proliferation and/or induce apoptosis. For example, preclinical studies have already yielded favorable results for a variety of cancers [38,39]. This category also includes FZD7-specific antibodies, which have been shown to be beneficial for Wilm’s tumor and HCC [40,41]. The third category includes peptides such as FZD2 binding proteins [42].

A number of compounds are undergoing clinical trials [43]. For example, OMP-18R5, a monoclonal antibody that targets the Frizzled receptors thereby preventing association with Wnt ligands, is being investigated for solid tumors (NCT01345201) [44,45]. OMP-54 F28 is a fusion protein of the FZD8 ligand-binding domain. It is again being investigated for solid tumors (NCT01608867) [46]. PRI-724 is a small molecule that inhibits interaction between β-catenin and CBP, whereas LGK974 is another small molecule that inhibits acyltransferase porcupine. Both are being investigated for different forms of cancers [46,47].

In conclusion, with the current era of advancements in anticancer therapeutics, therapies targeting the Wnt pathway do carry some significance [48]. However, it is worthwhile to note here that perturbations of the Wnt signalling pathway in normal cells can be lethal and it has been difficult to identify design specific inhibitory markers that could act on affected cells alone.

## Competing interests

The authors declare that they have no competing interests.

## Authors’ contributions

AS conceived the topic and was involved in drafting the initial manuscript. AKN, MZA, BI, SMS and HHK were involved in critically revising the manuscript, listed in decreasing order of their contributions. The authors have read and approved the manuscript. The authors did not receive any financial support/grant. All authors read and approved the final manuscript.
